# Contrasting roles of MERS-CoV and SARS-CoV-2 internal proteins in pathogenesis in mice

**DOI:** 10.1128/mbio.02476-23

**Published:** 2023-10-26

**Authors:** Lok-Yin Roy Wong, Abby Odle, Emma Luhmann, Douglas C. Wu, Yiquan Wang, Qi Wen Teo, Celeste Ptak, Alan Sariol, Shea Lowery, Matthias Mack, David K. Meyerholz, Nicholas C. Wu, Lilliana Radoshevich, Stanley Perlman

**Affiliations:** 1 Department of Microbiology, University of Iowa, Iowa City, Iowa, USA; 2 Department of Immunology, University of Iowa, Iowa City, Iowa, USA; 3 Department of Biochemistry, University of Illinois at Urbana-Champaign, Urbana, Illinois, USA; 4 Department of Internal Medicine, University Hospital Regensburg, Regensburg, Germany; 5 Department of Pathology, University of Iowa, Iowa City, Iowa, USA; 6 Carle Illinois College of Medicine, University of Illinois at Urbana-Champaign, Urbana, Illinois, USA; 7 Department of Pediatrics, University of Iowa, Iowa City, Iowa, USA; Johns Hopkins Bloomberg School of Public Health, Baltimore, Maryland, USA; University of Hong Kong, Pokfulam, Hong Kong, China

**Keywords:** MERS-CoV, pathogenesis, immune response, SARS-CoV-2

## Abstract

Betacoronaviruses encode an internal (I) gene via an alternative reading frame within the nucleocapsid gene, called ORF8b for Middle-East respiratory syndrome coronavirus (MERS-CoV) and ORF9b for severe acute respiratory syndrome coronavirus (SARS-CoV) and SARS-CoV-2. Previous reports suggested that proteins 8b and 9b are involved in evading multiple innate immune signaling pathways. However, their roles in mediating pathogenesis in infected animals have not been determined. In this study, we abrogated the expression of protein 8b in MERS-CoV and protein 9b in SARS-CoV-2. Using mouse models of MERS-CoV and SARS-CoV-2 infection, we found that MERS-CoV lacking protein 8b expression was more virulent, while SARS-CoV-2 lacking protein 9b expression was attenuated compared with the respective wild-type viruses. Upon further analysis, we detected increased levels of type I interferon and enhanced infiltration of immune cells to the lungs of mice infected with MERS-CoV lacking protein 8b expression. These data suggest that the I protein of MERS-CoV plays a role in limiting pathogenesis while that of SARS-CoV-2 enhances disease severity.

**IMPORTANCE:**

The function of betacoronavirus internal protein has been relatively understudied. The earliest report on the internal protein of mouse hepatitis virus suggested that the internal protein is a structural protein without significant functions in virus replication and virulence. However, the internal proteins of severe acute respiratory syndrome coronavirus (SARS-CoV), Middle-East respiratory syndrome coronavirus, and SARS-CoV-2 have been shown to evade immune responses. Despite the reported functions of the internal protein in these highly pathogenic human coronaviruses, its role in mediating pathogenesis in experimentally infected animals has not been characterized. Our data indicated that despite the similar genomic location and expression strategy of these internal proteins, their effects on virulence are vastly different and virus specific, highlighting the complexity between host-virus interaction and disease outcome.

## INTRODUCTION

*Coronaviridae* is a family of viruses of the order *Nidovirales*. Each genus of the *Coronaviridae* family encodes a set of distinct accessory proteins that are dispensable for virus replication but are important for modulating the host immune response (1). Members of the *Betacoronavirus* genus encode a small internal protein (I) within the open reading frame (ORF) of the nucleocapsid (N) protein that additionally goes by various virus-specific names, such as Middle-East respiratory syndrome coronavirus (MERS-CoV) ORF8b or severe acute respiratory syndrome coronavirus (SARS-CoV)/SARS-CoV-2 ORF9b (1, 2). The I protein shares the same transcription regulatory sequence (TRS) as the N gene and is translated through the +1 reading frame with respect to the N gene, possibly through a leaky ribosomal scanning mechanism (1, 2). The presence of the I protein is unique to betacoronaviruses; however, little is known about the functions of this protein. Previous studies suggested that the I proteins of highly pathogenic human coronaviruses contribute to immune evasion (3–6). Our previous study of MERS-CoV I protein (protein 8b) showed that it inhibits type I interferon (IFN-I) expression by impeding IRF3 activation (7). In a more recent study, Li et al. introduced MERS-CoV protein 8b into a recombinant neurotropic strain of mouse hepatitis virus (MHV) and showed that MHV expressing MERS-CoV protein 8b suppressed IFN-I expression in mice after infection, thereby increasing virus pathogenicity (7, 8). The I proteins (protein 9b) of SARS-CoV and SARS-CoV-2 have also been shown to impede IFN-I induction *in vitro* (3–6, 9).

In addition to suppressing IFN-I signaling, *in vitro* screening of SARS-CoV-2 ORF9b interacting partners revealed binding to host factors responsible for protein folding, intracellular vesicle trafficking, and endosomal recycling (9, 10). These studies suggest that the I proteins have diverse functions in affecting various host cell processes that may contribute to pathogenesis. However, the role of these proteins in infected animals has not been explored. For this purpose, we introduced two nonsense mutations into the internal proteins of mouse-adapted (MA) MERS-CoV (ORF8b) and SARS-CoV-2 (ORF9b). Both mouse-adapted viruses cause severe disease in mice. In the case of MERS-CoV, we infected mice in which mouse dipeptidyl peptidase 4 (DPP4) was partially replaced by human DPP4 (hDPP4-KI) since mice are naturally resistant to MERS-CoV (11–13). To our surprise, we found that genetic ablation of ORF8b caused enhanced virulence of MERS-CoV. However, deletion of ORF9b did not have the same effect but rather resulted in attenuation of SARS-CoV-2. Thus, two proteins with apparently similar functions displayed opposite effects on pathogenicity in the context of these two different pathogenic coronaviruses, suggesting virus-specific manifestations of disease outcome.

## RESULTS

### rMERS_MA_-Δ8b exacerbates disease in hDPP4-KI mice

**Fig 1:**
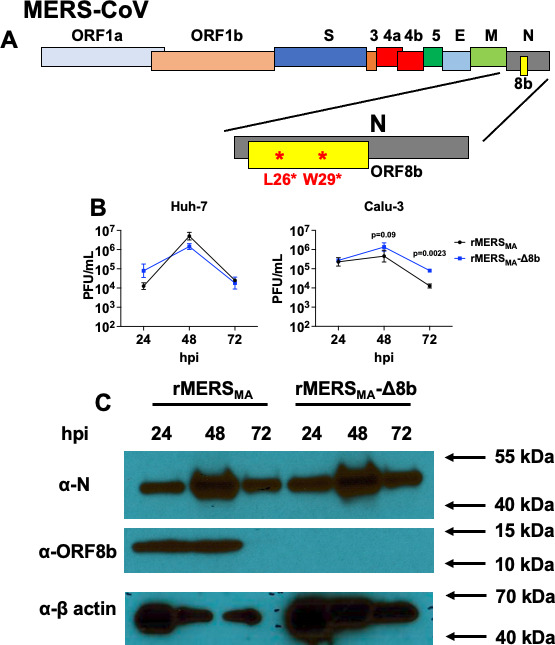
Generation and characterization of rMERS_MA_-Δ8b. (**A**) Schematic diagram showing the genome organization of MERS-CoV. The N protein region is amplified, and the location of the ORF8b protein is indicated. Two nonsense mutations (L26* and W29*) were introduced into the coding sequence of ORF8b resulting in a silent mutation in the corresponding N protein sequence. (**B**) Huh-7 (left panel) and Calu-3 (right panel) cells were infected with mouse-adapted MERS-CoV (rMERS_MA_) or MERS-CoV with the two nonsense mutations in the ORF8b protein (rMERS_MA_-Δ8b) at a multiplicity of infection (MOI) of 0.01. Infected cells were harvested by freeze thaw at the indicated time points, and the levels of infectious virus were determined by plaque assay. (**C**) Huh-7 cells were infected with rMERS_MA_ or rMERS_MA_-Δ8b at a MOI of 0.01. Infected cells were collected at the indicated time points for detection of the nucleocapsid (α-N) and ORF8b (α-ORF8b) protein expression. hpi, hour post infection. Data are representative of two independent experiments.

We previously abrogated ORF8b expression in the context of the EMC/2012 strain of MERS-CoV by introducing two nonsense mutations (L26* and W29*; * represents stop codon) into the ORF8b reading frame without affecting the N protein coding sequence (7). To investigate the role of ORF8b in mediating pathogenesis, we employed the same strategy in the context of the mouse-adapted strain of MERS-CoV (rMERS_MA_) (Fig. 1A). Similar to previous findings, ablation of ORF8b expression in the mouse-adapted background (rMERS_MA_-Δ8b) had minimal effects on virus replication in Huh-7 cells (7) but resulted in a modest but significant increase in rMERS_MA_-Δ8b-infected Calu-3 cells at 72 hpi (Fig. 1B). We confirmed protein 8b expression in rMERS_MA_-infected cells at 24 and 48 hpi but not at 72 hpi, possibly due to protein instability. While protein 8b expression was not detected in rMERS_MA_-Δ8b-infected cells, comparable N protein expression in cells infected with rMERS_MA_ and rMERS_MA_-Δ8b was detected (Fig. 1C).

**Fig 2:**
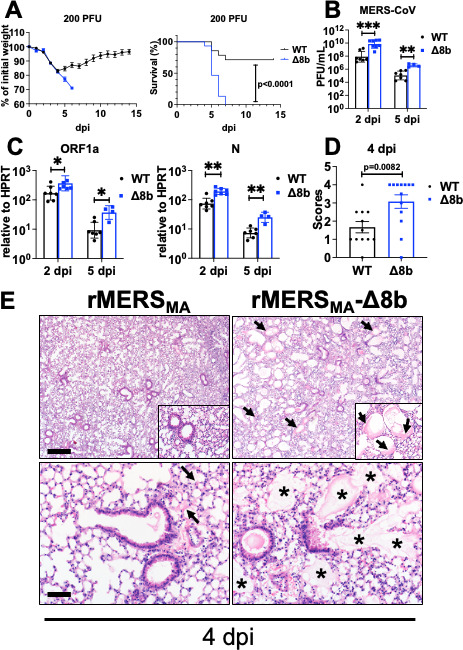
rMERS_MA_-Δ8b infection resulted in more severe disease in hDPP4-KI mice. hDPP4-KI mice were intranasally infected with 100–200 PFU of rMERS_MA_ or rMERS_MA_-Δ8b. (**A**) Percentages of initial weight (left panel) and survival (right panel) of infected mice are shown. (**B–E**) Lungs of the infected mice were harvested at the indicated time point. Infectious virus titers were determined by plaque assay (**B**), and genomic viral RNA levels (ORF1a, left panel; N, right panel) were determined by reverse transcription-quantitative real-time polymerase chain reaction (RT-qPCR) (**C**). Histopathological score for alveolar injury (edema and/or hyaline membranes) (**D**) and representative H&E stain (**E**) indicate extensive alveolar damage as shown by more prevalent edema (asterisks) and hyaline membrane (arrows) in rMERS_MA_-Δ8b-infected mice. Scale bar = 350 and 700 µm in the top and bottom panels, respectively. dpi, days post infection. Each point represents data obtained from an individual mouse. Data are pooled from two to three independent experiments.

We next investigated the role of protein 8b in mediating MERS-CoV virulence in mice, using hDPP4-KI mice as described (11). Infection with a sublethal dose (100–200 PFU) of rMERS_MA_ resulted in around 20% wt loss and modest lethality in hDPP4-KI mice. Unexpectedly, infection with the same dose of rMERS_MA_-Δ8b caused exacerbated weight loss and conferred significantly higher mortality in rMERS_MA_-Δ8b-infected mice compared with rMERS_MA_-infected mice (Fig. 2A). We detected significantly elevated levels of infectious virus and genomic viral RNA in the lungs of rMERS_MA_-Δ8b-infected mice compared with those of rMERS_MA_-infected mice at 2 and 5 dpi (Fig. 2B and C). Consistently, rMERS_MA_-Δ8b infection induced more prominent lung injury as evidenced by the formation of alveolar edema and/or hyaline membranes (Fig. 2D and E), suggesting that protein 8b functions to suppress virulence of MERS-CoV in hDPP4-KI mice.

**Fig 3:**
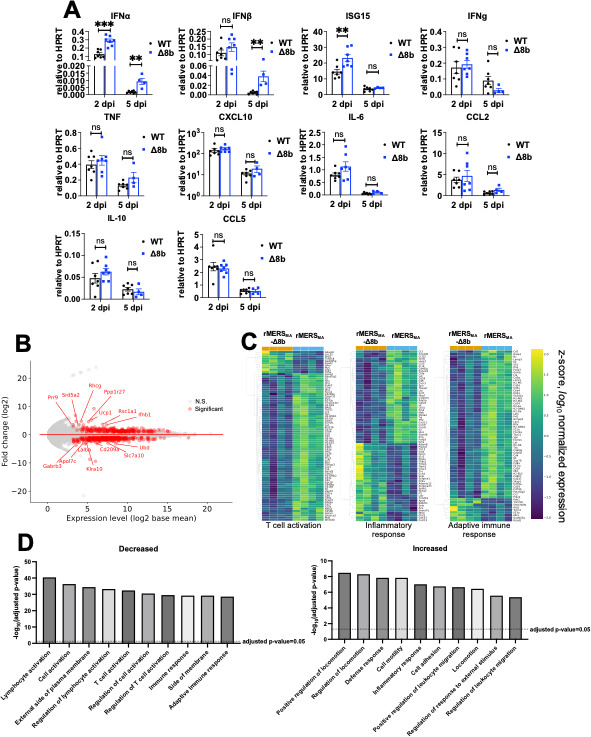
Transcriptomic analysis revealed distinct changes in rMERS_MA_-Δ8b-infected mouse lungs. (**A**) hDPP4-KI mice infected with 100–200 PFU of either rMERS_MA_ or rMERS_MA_-Δ8b virus were harvested at the indicated time points. Lungs were homogenized, and RNA was isolated for measurement of cytokine and chemokine levels by RT-qPCR. (**B**) Log_2_ fold change vs. log_2_ base mean for genes in the lung of rMERS_MA_-Δ8b-infected compared with rMERS_MA_-infected mice at 5 dpi are shown. Genes with adjusted *P* < 0.001 and log_2_ fold change > 2 are defined as differentially expressed genes and are highlighted in red. (**C**) Heat maps showing log_10_ z-score of selected genes involved in T cell activation (left panel), inflammatory response (middle panel) and adaptive immune response (right panel) at 5 dpi. (**D**) Ingenuity Pathway Analysis (Qiagen) was used to analyze altered biological pathways among differentially expressed genes (rMERS_MA_-Δ8b vs. rMERS_MA_) at 5 dpi. Pathways with adjusted *P* < 0.05 are considered significant. ns, not significant.

We next investigated changes in cytokine and chemokine expression in infected animals. Cytokine and chemokine transcripts levels in the lungs of rMERS_MA_- and rMERS_MA_-Δ8b-infected mice were mostly comparable except for IFN-I and ISG15 mRNA, which were upregulated in rMERS_MA_-Δ8b-infected mice, probably due to increased virus replication (Fig. 3A). To identify additional changes in gene expression in rMERS_MA_- and rMERS_MA_-Δ8b-infected mice, we performed bulk RNA sequencing (RNAseq) using lungs harvested at 5 dpi. Differentially expressed genes were defined as genes with adjusted *P* < 0.05 and log_2_ fold change > 2 (Fig. 3B). Genes related to T cell activation, inflammatory response, and adaptive immune response were distinctly regulated in rMERS_MA_-Δ8b-infected mice compared with rMERS_MA_-infected mice (Fig. 3C). To understand the differences between the rMERS_MA_- and rMERS_MA_-Δ8b-infected mice, we performed Ingenuity Pathway Analysis (IPA) to reveal biological pathways that were significantly altered. In comparison to rMERS_MA_-infected mice, mice infected with rMERS_MA_-Δ8b showed decreased expression of genes in pathways involved in lymphocyte activation, T cell activation, and adaptive immune responses. On another note, genes in pathways responsible for cellular locomotion, motility, and inflammatory response were elevated in rMERS_MA_-Δ8b-infected mice (Fig. 3D). These data are consistent with previous reports that prolonged/untimely innate immune responses, especially that those involving IFN-I contribute to enhanced inflammatory responses and inhibit the switch from innate immunity to adaptive immunity (14–19).

### rMERS_MA_-Δ8b infection enhances immune cell infiltration into the lungs of hDPP4-KI mice

**Fig 4:**
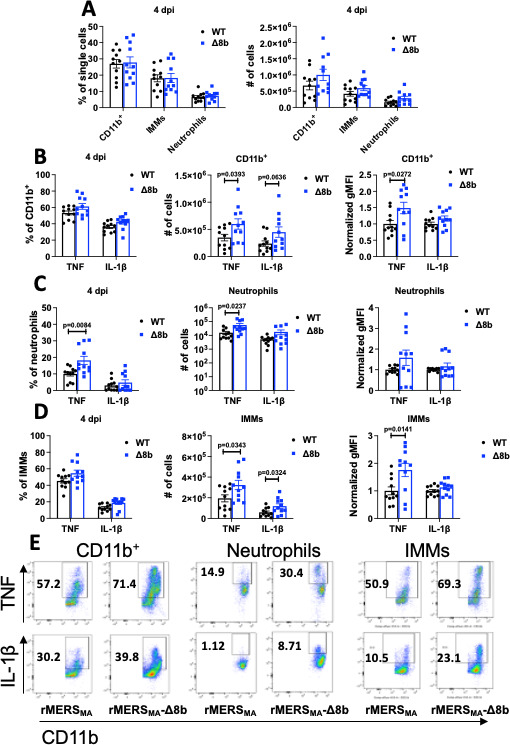
Increased immune cell activation and infiltration in rMERS_MA_-Δ8b-infected mice. hDPP4-KI mice were intranasally infected with 100–200 PFU of rMERS_MA_ or rMERS_MA_-Δ8b. The infected animals were sacrificed, and lungs were prepared for flow cytometric analysis at 4 dpi. (**A**) Frequency (left panel) and number (right panel) of total CD11b^+^ cells, inflammatory monocytes-macrophages, and neutrophils in the lung are shown. Frequency (left panel), number (middle panel), and normalized geometric mean fluorescence intensity (right panel) of total CD11b^+^ cells (**B**), neutrophils (**C**), and IMMs (**D**) expressing TNF and IL-1β are indicated. (**E**) Representative flow plots of TNF and IL-1β expression of total CD11b^+^ cells, neutrophils, and IMMs are shown. Each point represents data obtained from an individual mouse. Data are pooled from two to three independent experiments.

The RNA sequencing data revealed that genes involved in cellular locomotion, motility, and inflammatory response were upregulated in rMERS_MA_-Δ8b-infected mice (Fig. 3D). This prompted us to assess the levels of immune cell infiltration in the lungs of rMERS_MA_- and rMERS_MA_-Δ8b-infected mice (gating strategy shown in Fig. S1). We found similar frequency and number of CD11b^+^ cells, inflammatory monocytes-macrophages (IMMs), and neutrophils in the lungs of rMERS_MA_- and rMERS_MA_-Δ8b-infected mice. However, there were more CD11b^+^ cells and IMMs that produced TNF and IL-1β in the lungs of rMERS_MA_-Δ8b-infected mice. In addition, these cells were also more activated as shown by more robust TNF production (higher normalized geometric mean fluorescence intensity [gMFI]; Fig. 4). We also found increased frequency and number of neutrophils producing TNF in the lungs of rMERS_MA_-Δ8b-infected mice. These data suggest that rMERS_MA_-Δ8b induced a more robust inflammatory response in infected animals that likely contributed to enhanced pathogenicity of rMERS_MA_-Δ8b.

Since these results suggested key roles for IFN-I and infiltrating IMMs in mediating the enhanced pathogenicity of rMERS_MA_-Δ8b, we used antibodies to either block IFN-I signaling or deplete IMMs. However, neither of these interventions provided any protection against rMERS_MA_-Δ8b infection, suggesting that in addition to possible contributions of IFN-I or infiltrating IMM, other inflammatory factors play a role in pathogenicity (Fig. S2).

### Virus-specific role of ORF8b

A recent study showed that the introduction of protein 8b into a neurovirulent strain of murine coronavirus (MHV) resulted in enhanced disease in mice (20). Independently, we generated two MHV recombinants in which ORF8b (rMHV-8b) or ORF8b with L26* and W29* mutations (rMHV-8b*) was inserted in place of MHV ORF4. Western blot analysis confirmed the expression of protein 8b in cells infected with rMHV-8b (Fig. S3). Consistent with the previous report (20), young male C57BL/6 mice infected with rMHV-8b showed more severe clinical disease and reduced survival compared with those infected with rMHV or rMHV-8b* (Fig. S3). These data indicate that in the context of a heterologous infection, the presence of ORF8b enhanced virulence, as opposed to the case of MERS-CoV, where ORF8b expression resulted in attenuation, suggesting a virus-specific role of ORF8b.

Since protein 8b had immunoevasive properties in the context of MHV (20), we next queried whether expression elsewhere in the MERS-CoV genome would reverse the apparent disease-ameliorating effects observed when encoded within the N gene. To examine this possibility, we constructed two recombinant MERS-CoVs by introducing ORF8b (Δ8b-8b) or ORF8b with L26* and W29* mutations (Δ8b-8b*) to partially replace ORF5 of rMERS_MA_-Δ8b (Fig. S4A). The first 13 amino acids of ORF5 were retained because complete deletion of ORF5 results in enhanced virulence. Protein 8b was only expressed in cells infected with Δ8b-8b but not in those infected with Δ8b-8b* (Fig. S4B). However, mice infected with Δ8b-8b did not show a significant increase in survival compared with that infected with Δ8b-8b* and the levels of infectious virus were similar in the lungs of Δ8b-8b- and Δ8b-8b*-infected mice (Fig. S4C). These data suggest that either the level of protein 8b in Δ8b-8b-infected cells was insufficient to revert the enhanced virulence or that the genomic location of ORF8b plays a role in determining pathogenicity of the virus.

### SARS-CoV-2-Δ9b causes attenuated disease in mice

**Fig 5:**
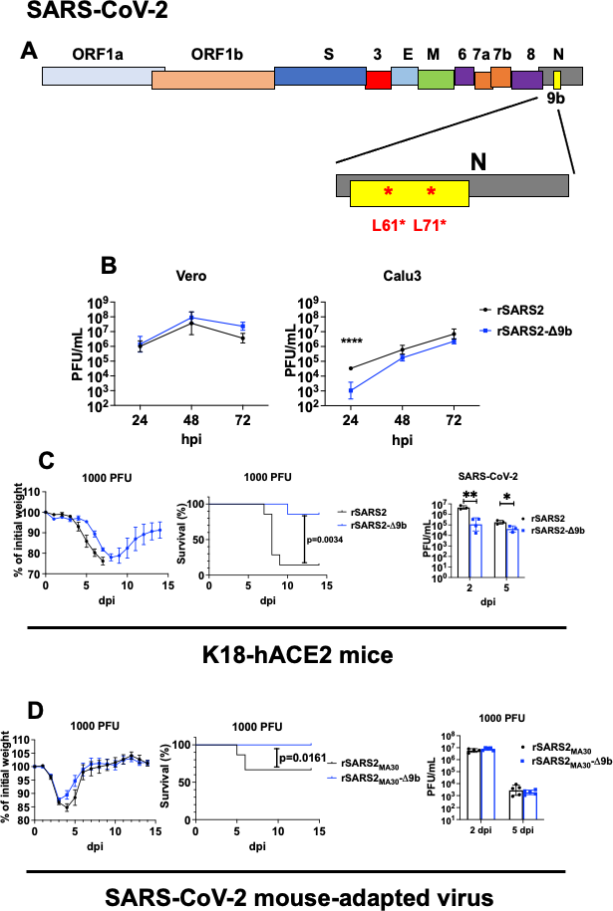
rSARS2-Δ9b is attenuated in mice. (**A**) Schematic diagram showing the genome organization of SARS-CoV-2. The N protein region is amplified, and the location of the ORF9b protein is indicated. Two nonsense mutations (L61* and L71*) were introduced into the coding sequence of ORF9b that result in a silent mutation in the corresponding N protein sequence. (**B**) Vero (left panel) and Calu-3 (right panel) cells were infected with SARS-CoV-2 (rSARS2) or SARS-CoV-2 with the two nonsense mutations in the ORF9b protein (rSARS2-Δ9b) in the Wuhan-Hu-1 background at a multiplicity of infection (MOI) of 0.01. Infected cells were harvested by freeze thaw at the indicated time points, and the levels of infectious virus were determined by plaque assay. (**C**) K18-hACE2 transgenic mice were intranasally infected with 1,000 PFU rSARS2 and rSARS2-Δ9b. Percentages of initial weight (left panel), survival (middle panel), and infectious virus titers in the lungs at the indicated time point (right panel) are shown. (**D**) C57BL/6 mice were intranasally infected with 1,000 PFU of rSARS2_MA_ or rSARS2_MA_-Δ9b. Percentages of initial weight (left panel), survival (middle panel), and infectious virus titers in the lungs at the indicated time point (right panel) are shown. Each point represents data obtained from an individual mouse. Data are pooled from two to three independent experiments.

Given these results, we next examined the role of the internal protein in the context of another pathogenic human CoV, SARS-CoV-2. Like MERS-CoV-specific protein 8b, SARS-CoV-2 protein 9b is believed to have immunoevasive properties. We introduced two nonsense mutations (L61* and L71*; * represents stop codon) into the Wuhan-Hu-1 background (rSARS2) and generated SARS-CoV-2 that encodes two stop codons within the ORF9b reading frame (rSARS2-Δ9b) (Fig. 5A). Replication kinetics between rSARS2 and rSARS2-Δ9b in Vero cells, which are unable to mount an interferon response, were comparable. However, there was a significant reduction in rSARS2-Δ9b compared with rSARS2 titers at 24 h after infection of Calu-3 cells, which are interferon competent (Fig. 5B). We next infected K18-hACE2 mice with equal amounts of either rSARS2 or rSARS2-Δ9b. We observed reduced weight loss, faster recovery, and significant reduction in mortality in mice infected with rSARS2-Δ9b (Fig. 5C). The levels of infectious virus were also significantly lower in the lungs of rSARS2-Δ9b-infected compared with rSARS2-infected K18-hACE2 mice. We did not detect infectious virus in the brains of any of the infected animals. We next introduced two stop codons into the ORF9b gene of mouse-adapted SARS-CoV-2 (rSARS2_MA30_-Δ9b) (21) and infected C57BL/6 mice with 1,000 PFU of mouse-adapted WT virus (rSARS2_MA30_) or rSARS2_MA30_-Δ9b. Similar to the infected K18-hACE2 mice, we observed reduced weight loss and increased survival in rSARS2_MA30_-Δ9b-infected mice (Fig. 5D). We did not observe significant changes in infectious virus titers in the lungs of the infected animals at 2 and 5 dpi (Fig. 5D), consistent with the relatively small effects on clinical disease that were observed. In a similar vein, we did not observe major differences in lung pathology of mice infected with either virus (Fig. S5A). To probe the inflammatory response, we measured cytokine and chemokine levels in the lungs of rSARS2_MA30_- and rSARS2_MA30_-Δ9b-infected mice at 2 dpi (Fig. S5B). We observed similar expression levels for most of the cytokines and chemokines assayed in these mice, except modest but significant increase in IL-6 and ISG15 in rSARS2_MA30_-Δ9b-infected mice. Collectively, these results suggested that SARS-CoV-2 ORF9b is a virulence factor, and in its absence, the host immune response is modestly increased.

### Mutations in SARS-CoV-2 VOCs that enhanced 9b expression did not cause significant changes in virulence

**Fig 6:**
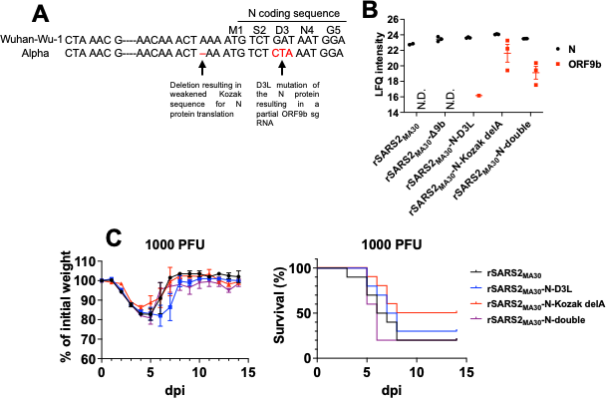
Mutations in VOC that enhanced ORF9b protein expression did not result in significant change in virulence. (**A**) Schematic diagram showing the mutations (highlighted in red) in the Kozak and protein coding sequence of the N gene that were proposed to enhance ORF9b protein levels. (**B**) Calu-3 cells were infected with SARS-CoV-2 mouse-adapted virus (rSARS2_MA30_), SARS-CoV-2 mouse-adapted virus encoding either a glutamate to leucine substitution at the third amino acid of the N protein (rSARS2_MA30_-N-D3L) or an adenosine deletion in the Kozak sequence of the N gene (rSARS2_MA30_-N-Kozak delA), or a mutant virus that encodes both mutations (rSARS2_MA30_-N-double) at a multiplicity of infection of 0.01. Infected cells were harvested at the indicated time points for mass spectrometry. Log_2_-transformed label-free quantification (LFQ) intensity values of the N protein (black) and ORF9b (red) in three biological replicates are shown (except for rSARS2_MA30_-infected samples, where one replicate was excluded as being an outlier in intensity). ORF9b peptides were not detected for rSARS2_MA30_- and rSARS2_MA30_-Δ9b-infected samples (N.D., not detected). ORF9b peptides were only detected in one of the three replicates of rSARS2_MA30_-N-D3L-infected samples as shown. (**C**) C57BL/6 mice were intranasally inoculated with 1,000 PFU of rSARS2_MA30_, rSARS2_MA30_-N-D3L, rSARS2_MA30_-N-Kozak delA, or rSARS2_MA30_-N-double. Percentages of initial weight (top panel) and survival (bottom panel) of infected mice are shown. Each point in 6A represents data obtained from an individual mouse. Data are pooled from two to three independent experiments.

Since several reports showed that some SARS-CoV-2 VOCs evolved to express higher levels of ORF9b (22), we next investigated whether these changes resulted in more profound effects on pathogenesis in mice. Specifically, the alpha and delta variants of SARS-CoV-2 evolved to encode an adenosine deletion that weakens the Kozak sequence of the N protein, resulting in reduced N protein translation and enhanced protein 9b expression by leaky ribosomal scanning (23) (Fig. 6A). Also, an aspartic acid to leucine (D3L) substitution in the N protein, found only in the alpha variant, was predicted to introduce an enhanced TRS for ORF9b (22) (Fig. 6A). These two mutations emerged during SARS-CoV-2 evolution and could contribute to enhanced innate immune evasion of some VOCs . To investigate the effects of these two mutations in mediating pathogenesis, we introduced the adenosine deletion (rSARS2_MA30_-N-Kozak delA) and the D3L substitution (rSARS2_MA30_-N-D3L) individually, or in combination (rSARS2_MA30_-N-double) into the mouse-adapted virus. We used label-free quantitative mass spectrometry (LC-MS/MS) to detect changes in 9b protein expression since an anti-9b antibody is not available. N protein expression was uniformly observed in all samples. A total of nine peptides were detected for protein 9b. A list of the nine peptides detected for protein 9b and the spectral count and intensity of each detected peptide for individual biological replicates are shown in Table S1. We also detected high levels of 9b peptides in rSARS2_MA30_-N-Kozak delA- and rSARS2_MA30_-N-double-infected samples (Fig. 6B). However, we were only able to detect one of the nine peptides at low levels in cells infected with rSARS2_MA30_- or rSARS2_MA30_-N-D3L (Table S1). This suggests that the levels of protein 9b in rSARS2_MA30_-infected samples were low and that the levels of protein 9b were elevated in rSARS2_MA30_-N-Kozak delA- and rSARS2_MA30_-N-double-infected samples, allowing for detection. We next infected C57BL/6 mice separately with equal amounts of rSARS2_MA30_, rSARS2_MA30_-N-D3L, rSARS2_MA30_-N-Kozak delA, and rSARS2_MA30_-N-double. We did not observe evidence of increased pathogenicity in mice infected with viruses expressing more protein 9b (Fig. 6C), suggesting that, even though these mutations found in the VOCs resulted in enhanced ORF9b expression, the effect of this augmented expression was minimal, at least in mice.

### Structural comparison of SARS-CoV, SARS-CoV-2, and MERS-CoV internal protein

**Fig 7:**
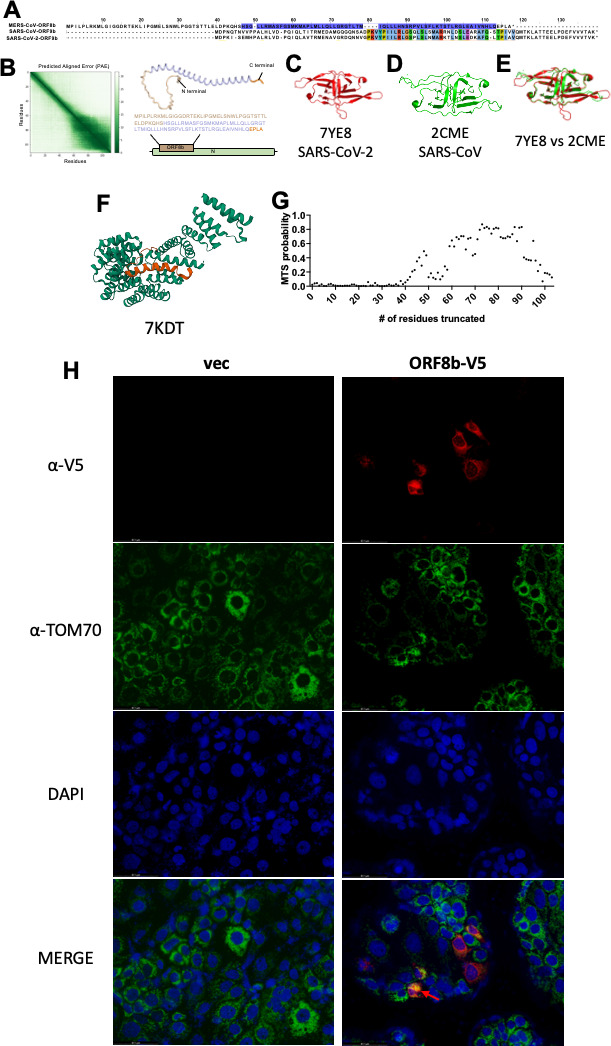
Sequence and structural analysis of MERS-CoV, SARS-CoV, and SARS-CoV-2 internal proteins. (**A**) Sequence alignment of MERS-CoV ORF8b, SARS-CoV ORF9b, and SARS-CoV-2 ORF9b reveals a low degree of sequence identity between MERS-CoV ORF8b and SARS-CoV or SARS-CoV-2 ORF9b. SARS-CoV and SARS-CoV-2 ORF9b show high level of sequence similarity. (**B**) Prediction of the structure of MERS-CoV ORF8b with AlphaFold (24). The predicted aligned error (left panel) and the corresponding amino acids that constitute the different parts of the structure are coded with the same color. MERS-CoV ORF8b consists of flexible N- and C-termini with a long central alpha helix. Published structures of SARS-CoV-2 (**C**) and SARS-CoV (**D**) ORF9b. Both SARS-CoV-2 (PDB 7YE8) and SARS-CoV (PDB 2CME) ORF9b exist as homodimer in these published structures. (**E**) The dimeric structures of SARS-CoV-2 (**C**) and SARS-CoV (**D**) protein 9b were superimposed and demonstrate structural similarity. (**F**) Published structure of SARS-CoV-2 ORF9b complexed with TOM70 (PDB 7KDT). (**G**) Prediction of mitochondrial targeting signal probability using TargetP server. Serially truncated sequence of MERS-CoV ORF8b from the N-terminus was subject to analysis for the probability of encoding an internal MTS. The region with highest MTS probability (residues 60–90) is located within the alpha helix (Fig. 7B). (**H**) Huh-7 cells were transfected with empty vector (vec) or a plasmid expressing MERS-CoV ORF8b with a C-terminal V5 tag (ORF8b-V5). Cells were fixed and stained at 48 h post transfection. MERS-CoV ORF8b (red), TOM70 (green), and DAPI (blue) were visualized. Moderate co-localization of MERS-CoV ORF8b and TOM70 is indicated by the red arrow.

To further understand the role of the different internal proteins, we compared the sequences and structures of the internal proteins of the three highly pathogenic human coronaviruses. MERS-CoV protein 8b shows a low degree of sequence similarity with protein 9b of both SARS-CoV and SARS-CoV-2 (Fig. 7A). Since there are no published structures for MERS-CoV protein 8b, we predicted its structure using AlphaFold (24). From the predicted structure, protein 8b is composed of an N-terminal linker region, an internal alpha helix that spans from residue H47 to Q108, and a C-terminal tail (Fig. 7B). This is structurally distinct from the published structures of SARS-CoV and SARS-CoV-2 protein 9b (Fig. 7C and D). SARS-CoV and SARS-CoV-2 protein 9b share extensive sequence and structural similarity in protomeric (Fig. S6A) and dimeric formers (Fig. 7E), which are mainly composed of beta sheets with loop regions (9, 25). To verify the accuracy of AlphaFold, the structures of SARS-CoV and SARS-CoV-2 protein 9b were also predicted and superimposed with the respective published protomeric structures (Fig. S6B and C). The predicted structures share high-degree similarity to the published structure, suggesting that AlphaFold provided accurate structural prediction. However, when SARS-CoV-2 protein 9b is complexed with the mitochondrial adaptor protein TOM70, its structure is primarily alpha-helical (Fig. 7F, orange ribbon; corresponding amino acid residues are highlighted in Fig. 7A), suggesting that SARS-CoV-2 protein 9b exists as an alpha helix when it interacts with other proteins (26). Since SARS-CoV-2 protein 9b was predicted to encode an internal mitochondrial targeting signal (MTS), we interrogated the sequence of MERS-CoV protein 8b for TargetP MTS prediction analysis (27). As shown in Fig. 7G, the region of protein 8b with high MTS probability is buried within the alpha helix region (Fig. 7A, highlighted in purple font; Fig. 7B, highlighted in lilac ribbon and font). To investigate the interaction between protein 8b and TOM70, we expressed ORF8b with a V5 tag and stained for TOM70 expression. We found that protein 8b showed signs of colocalization with TOM70 (Pearson’s coefficient of 0.59; Fig. 7H, red arrow), suggesting a moderate correlation between TOM70 and ORF8b in some cells.

### MERS-CoV ORF8b and SARS-CoV-2 ORF9b do not affect replication in the upper airway

A notable difference between MERS-CoV and SARS-CoV-2 is the greater transmissibility of the latter, which is conferred in part by its ability to replicate to high titers in the upper airway. MERS-CoV lacking ORF8b expression grows to higher titers in the lungs. To determine whether this also might impact titers in the upper respiratory tract, and by extension, transmissibility, we infected hDPP4-KI mice with rMERS_MA_ and rMERS_MA_-Δ8b and as a control, C57BL/6 mice with rSARS2_MA30_ and rSARS2_MA30_-Δ9b. We measured the levels of infectious virus in the nasal turbinates (Fig. S7). We did not observe any significant difference in hDPP4-KI mice infected with rMERS_MA_ or rMERS_MA_-Δ8b and C57BL/6 mice infected with rSARS2_MA30_ or rSARS2_MA30_-Δ9b, suggesting that the Internal protein of MERS-CoV and SARS-CoV-2 did not contribute to transmission.

## DISCUSSION

In this study, we showed that MERS-CoV lacking protein 8b expression became more virulent while SARS-CoV-2 lacking protein 9b expression was attenuated relative to their wild-type counterparts. Proteins 8b and 9b share similar expression strategy and genomic location in their respective viruses, and the two proteins have been shown to antagonize IFN-I expression in tissue culture models (3, 4, 7). It is curious that MERS-CoV and SARS-CoV-2 displayed opposite effects on virulence when these IFN-I antagonists, protein 8b and protein 9b for MERS-CoV and SARS-CoV-2, respectively, were removed, indicating that viral proteins with similar functions in overexpression assays can differentially contribute to pathogenesis in a virus-specific context. This is consistent with the observation that the presence of protein 8b in the context of MHV infection resulted in more severe disease (20) (Fig. 1), while protein 8b expression in the context of MERS-CoV infection attenuated the infection (Fig. 2A, rMERS_MA_ vs. rMERS_MA_-Δ8b). One possibility is that protein 8b in the context of MERS-CoV interacts with another MERS-CoV protein(s), hence sequestering other viral proteins to impair their functions. Other possible scenarios, including dysregulated/delayed IFN-I responses induced by different coronaviruses and potential proinflammatory functions of protein 8b and protein 9b in addition to IFN-I antagonism, remain to be investigated to fully elucidate the contribution of the two proteins to virulence and pathogenesis. Also, the introduction of two stop codons does not eliminate the possibility that a truncated, N-terminal part of protein 8b could still be expressed.

The ameliorating role of protein 8b was unexpected but is not unprecedented in MERS-CoV-infected mice. Deletion of ORF5 from MERS-CoV, another gene with immunoevasive function, also resulted in increased virulence (28). Virus replication increased in the absence of either protein 5 or protein 8b, with evidence of a delayed and dysregulated innate immune response or enhanced inflammation including IFN-I expression or IFN-I/ISG expression observed, respectively. There is precedent in coronavirus-infected mice and patients for a dysregulated IFN response contributing to increased virus replication and disease severity (16, 18, 19). In mice, a delayed IFN-I response resulted in augmented lung entry of IMMs and either antibodies that blocked IFN-I function or IMM entry into the lungs enhanced survival (14, 15). Our results showed that neither intervention diminished lethality after rMERS_MA_-Δ8b infection (Fig. S1), suggesting that inflammatory factors other than IFN-I or IMM infiltration play a role in pathogenicity. We also recognize that it is possible that protein 8b (and protein 5) has novel, unrecognized functions in MERS-CoV-infected cells and animals.

Collectively, these results suggest that the idea that coronaviruses encode accessory proteins for immunoevasion to attain optimal virus replication and dissemination may need to be nuanced in the context of MERS-CoV. The fact that human and camel MERS-CoV nearly invariably encode protein 8b and 5 suggest that the presence of these two accessory proteins provide prominent evolutionary advantage unlike other MERS-CoV accessory proteins, such as ORFs 3, 4a, and 4b, which are variably deleted (29). Further studies on how MERS-CoV accessory proteins interact with different hosts may provide insights into differential *in vivo* requirements that allow the virus to thrive in different hosts and transmit efficiently.

Previous reports have indicated a dimeric structure of SARS-CoV and SARS-CoV-2 ORF9b with multiple beta sheets when crystalized in the absence of interacting proteins (9, 25). However, when complexed with TOM70, SARS-CoV-2 ORF9b exists as a protomer with an alpha helix structure (26). This structure resembles the predicted protomeric structure of MERS-CoV ORF8b (Fig. 7B). In addition, both SARS-CoV-2 protein 9b and MERS-CoV protein 8b encode internal MTS in the form of helices (26) (Fig. 7F). These features are consistent with the binding preference of TOM70 C-terminal tetratricopeptide repeat (TPR) motif, suggesting potential interaction between MERS-CoV protein 8b and TOM70. TOM70 has been reported to interact with heat shock proteins (Hsp) 70 and 90 through its N-terminal TPR motif to facilitate import of mitochondrial proteins (30). TOM70 has also been shown to regulate transcription of mitochondrial proteins and mitochondrial biogenesis (31). The binding of SARS-CoV-2 protein 9b was reported to impair Hsp binding to TOM70 (26), suggesting that SARS-CoV-2 protein 9b may have additional roles in causing mitochondrial dysfunction and mitophagy. In addition, MERS-CoV protein 8b was reported to interact with Hsp to impair IFN-I expression (7). Whether the IFN-I suppressive function of protein 8b is mediated through TOM70 and the involvement of MERS-CoV protein 8b in mitochondrial dysfunction remain to be investigated. Furthermore, although SARS-CoV protein 9b shares extensive sequence and structural similarities with SARS-CoV-2 protein 9b, whether SARS-CoV protein 9b interacts with TOM70 requires further investigation. If SARS-CoV protein 9b and MERS-CoV protein 8b interactions with TOM70 were confirmed, it would suggest that different highly pathogenic human coronaviruses encode accessory proteins of diverse sequences yet with convergent molecular functions, despite having different roles in pathogenesis.

Several reports have suggested that SARS-CoV-2 evolved to enhance innate immune evasion. These reports suggested that such an enhanced evasion during evolution was a consequence of accumulation of mutations that resulted in increased expression of accessory proteins (22, 23). In particular, SARS-CoV-2 Alpha and Delta variant encode mutations that enhanced protein 9b expression (22). The increased expression of protein 9b was suggested to partially mediate the enhanced innate immune evasion (22, 23). We showed that 9b peptides were detected in abundance from samples infected with the rSARS2_MA30_-N-Kozak delA and rSARS2_MA30_-N-double viruses, but only at low levels in samples infected with rSARS2_MA30_ and rSARS2_MA30_-N-D3L viruses, indicating that the adenosine deletion in the Kozak sequence of the N gene is the major factor driving increased protein 9b expression (Fig. 6A). However, we did not identify significant changes in clinical disease in mice infected with SARS-CoV-2 encoding these mutations compared with those infected with rSARS2_MA30_ (Fig. 6B). This suggests that the varying levels of protein 9b in the reported VOCs did not cause any significant changes in SARS-CoV-2 virulence, at least in infected mice. This is also consistent with our results that rSARS2_MA30_-Δ9b virus only showed minor attenuation (Fig. 5C and D). The enhanced innate immune evasion observed in the VOCs could potentially be a net effect of all the changes identified.

Overall, we demonstrated the opposing effects on virulence when protein 8b or 9b expression was abrogated in MERS-CoV or SARS-CoV-2, respectively. The results also further illustrated the unique properties that putative immunoevasive proteins have in the context of MERS-CoV and suggest that coronavirus accessory proteins of similar functions *in vitro* may cause drastic changes *in vivo* in a virus-specific manner. These results reinforce the notion that the function of viral proteins needs to be validated in the context of infectious virus to accurately reflect the actual function of the protein being investigated.

## MATERIALS AND METHODS

### Mice, cells, and viruses

Human DPP4-KI and human K18-ACE2 transgenic mice were generated and propagated as described previously (11, 32). Both male and female hDPP4-KI mice of 6–10 weeks of age were used in this study for MERS-CoV. rMERS_MA_ was generated by introducing mouse-adapted mutation into the EMC strain by reverse genetics. rMERS_MA_-Δ8b was generated as described below. Male and female K18-hACE2 mice of 8–12 weeks of age were used for SARS-CoV-2 infection with viruses derived on the Wuhan-Hu-1 background. Ten- to 16-week-old C57BL/6 mice were obtained from Charles River Laboratories for infection with recombinant SARS-CoV-2 derived on a mouse-adapted background (21). Five- to 7-week-old male C57BL/6 mice were used in this study for MHV infection. Mice were maintained in the Animal Care Unit at the University of Iowa under standard conditions of dark/light cycle, ambient temperature, and humidity. Calu-3 2B4 cells were grown in Dulbecco’s modified Eagle’s medium (DMEM, GIBCO, Grand Island, NY) supplemented with 20% fetal bovine serum (FBS). Vero E6 (ATCC CRL-1586), Vero 81 (ATCC CCL-81), and Huh-7 cells were grown in DMEM supplemented with 10% FBS. MERS-CoV, SARS-CoV-2, and a recombinant version of the neuroattenuated J2.2-V-1 variant of JHMV (MHV) were propagated in Huh-7, Calu-3, and 17Cl-1 cells, respectively. Virus titers of MERS-CoV, SARS-CoV-2, and MHV-J2.2 were determined in Vero 81, Vero E6, and HeLa-MHVR cells, respectively. Studies involving the use of recombinant viruses have been approved by the Institutional Biosafety Committee of the University of Iowa. The enhanced virulence of rMERS_MA_-Δ8b was reported to the NIH, and no concerns were raised.

### Mouse infection

Mice were anesthetized with ketamine-xylazine and infected intranasally with the indicated amount of virus in a total volume of 50 µL of DMEM. Animal weights and clinical disease were monitored daily. For intracranial infection, mice were anesthetized with ketamine-xylazine. Mice were inoculated with 750 PFU of MHV in 30 µL of DMEM. Clinical scoring for MHV infection was based on the following criteria: 0, asymptomatic; 1, limp tail, mild hunching; 2, wobbly gait with mild righting difficulty, hunching; 3, hind-limb paresis and extreme righting difficulty; 4, hind-limb paralysis; and 5, moribund. All experiments with MERS-CoV and SARS-CoV-2 were performed in a biosafety level 3 (BSL3) Laboratory at the University of Iowa.

### Virus titer by plaque assay

At the indicated times, cells were frozen and mice were euthanized and transcardially perfused with PBS. Organs were harvested and homogenized, while cells were thawed prior to clarification by centrifugation and titering. Virus or tissue homogenate supernatants were serially diluted in DMEM. Twelve-well plates of Vero E6 (for SARS-CoV-2) or Vero 81 cells (for MERS-CoV) or HeLa-MVR (for MHV) were inoculated at 37°C in 5% CO_2_ for 1 h and gently rocked every 15 min. For MERS-CoV and SARS-CoV-2, inocula were removed and plates were overlaid with 0.6% agarose containing 2% FBS. Cells were incubated for 3 days. After 3 days, cells were fixed with 10% formaldehyde for 20 min and overlays were removed, and plaques visualized by staining with 0.1% crystal violet. For MHV, DMEM supplemented with 10% FBS was added to the well and incubated for 16 h. After 16 h, the supernatant was removed and plates were overlaid with 0.6% agarose containing 2% FBS and 0.1% neutral red and incubated for 4 h. Plaques were counted without further treatment after 4 h of incubation. Viral titers were quantified as PFU/mL tissue.

### Lung histology

Mice were anesthetized by intraperitoneal injection of ketamine-xylazine. Lungs were transcardially perfused with 10 mL PBS. Lungs were removed, fixed in zinc formalin, and paraffin embedded. Sections were prepared, stained with H&E, and scored in a blinded fashion (33) for edema and inflammation with scores of 0, 1, 2, 3, and 4 representing lung areas with 0%, less than 25%, 26%–50%, 51%–75%, and more than 75% detectable lung injury (edema/hyaline membranes) and inflammation, respectively.

### Lung cell preparation and antibodies for flow cytometric analysis

Animals were anesthetized with ketamine-xylazine and perfused transcardially with 10 mL PBS. Lungs were removed, minced, and digested in HBSS buffer consisting of 2% fetal calf serum, 25 mM HEPES, 1 mg/mL collagenase D (Roche), and 0.1 mg/mL DNase (Roche) at 37°C for 30 min. Single-cell suspensions were prepared by passage through a 70-µM cell strainer. Cells were enumerated with a Scepter 2.0 cell counter (MilliporeSigma). Cells were then washed and blocked with 1 µg α-CD16/α-CD32 (2.4G2) antibody at 4°C for 20 min and surface stained with the following antibodies at 4°C for 30 min: BV570 α-CD45 (30-F11; BioLegend); eFluor 450 α-CD11b (M1/70; eBioscience); PE α-Siglec F (S17007L; BioLegend); APC α-Ly6G (1A8; BioLegend); APC/eFluor 780 α-Ly6C (HK1.4; BioLegend); and LIVE/DEAD Fixable Blue Dead Cell Stain Kit (Invitrogen). For intracellular cytokine staining (ICS), lymphocytes were cultured in 96-well dishes at 37°C for 5–6 h in the presence of brefeldin A (BD Biosciences). Cells were then labeled for cell surface markers, fixed/permeabilized with Cytofix/Cytoperm Solution (BD Biosciences), and labeled with PerCP/eFluor 710 α-IL-1β (NJTEN3; eBioscience) and PE/Cy7 α-TNF (MP6-XT22; eBioscience) antibody. All flow cytometry data were acquired using a BD FACSVerse and analyzed with FlowJo software. Geometric mean fluorescence intensity of TNF- and IL-1β-positive cells (gMFI) was normalized to the average gMFI of TNF- and IL-1β-positive cells of the rMERS_MA_ samples, respectively, to obtain normalized gMFI. α-MERS-CoV ORF8b antibody was a gift from Dr. Dongyan Jin at the School of Biomedical Sciences, The University of Hong Kong, Hong Kong (7). α-IFNAR antibody (clone MAR1-5A3), mouse IgG isotype control (clone MOPC-21), and rat IgG isotype control (Cat.# BE0094) were purchased from BioXcell. α-CCR2 antibody (clone MC-21) was used in the IMM depletion studies (34).

### RNA isolation and RT-qPCR

Cells were harvested and lung tissue were homogenized in TRIzol (Invitrogen) for RNA isolation as specified by the manufacturer’s protocol. Isolated RNA was subject to DNase I treatment (Invitrogen) and reverse transcribed using the SuperScript IV First-Strand Synthesis System (Invitrogen). mRNA levels were determined after normalizing with HPRT by the ΔCt method. Specific primer sets used for qPCR were previously described (15, 35).

### Total protein extraction and western blotting

Huh-7 cells infected with MERS-CoV were lysed with RIPA buffer (Invitrogen) at the indicated time points and mixed with gel-loading dye for 10 min at 95°C before electrophoresis. Cell lysates were resolved by SDS-PAGE. Specific protein bands were revealed by the following primary antibodies: rabbit α-ORF8b polyclonal antibody (7) and mouse α-N monoclonal antibody (40068-MM10; SinoBiological).

### Immunofluorescence

Huh-7 cells seeded on cover slips were transfected with a plasmid expressing MERS-CoV ORF8b with a C-terminal V5 tag. At 48 h post transfection, cells were washed with PBS and fixed with 4% paraformaldehyde for 15 min at room temperature. Cells were washed three times with PBS and permeabilized in 0.75% of Triton X-100 for 20 min. Permeabilized cells were blocked with 1% BSA for 1 h at room temperature and stained with primary antibodies (α-V5, 1:100 dilution, Invitrogen Cat.# R960-25; α-TOM70, 1:100 dilution, Abcam Cat.# ab289977) at 4 degrees overnight. Cells were washed three times with PBS the next day and stained with secondary antibodies at room temperature for 1 h. Cells on cover slips were washed three times with PBS and mounted with Vectashield (Cat.# H-1800) on slides for imaging.

### Generation of MERS-CoV and SARS-CoV-2 mutant BACs

MERS-CoV and SARS-CoV-2 mutant BACs were generated using lambda red recombination with I-SceI homing endonuclease as described previously (36). In brief, forward and reverse primers containing sequences complementary to the area of interest of MERS-CoV/SARS-CoV-2 and the desired mutation, followed by a sequence complementary to the target plasmid (pEP-KanS), were designed. PCR fragments with overlapping ends carrying MERS-CoV/SARS-CoV-2 sequences flanking a kanamycin resistance marker were amplified with the indicated primers using pEP-KanS as template. PCR fragments were purified using a PureLink Quick Gel Extraction and PCR Purification Combo Kit (Invitrogen). Purified PCR fragments were electroporated into the GS1783 strain of *Escherichia coli* carrying the MERS-CoV/SARS-CoV-2 BAC. Successful recombinants were selected based on kanamycin resistance. The kanamycin markers were removed by arabinose induction of I-SceI cleavage followed by homologous recombination of the overlapping ends. Successful recombinants were selected by replica plating for the loss of kanamycin resistance. Recombinants were purified and sequenced to confirm the introduction of the mutation into the BAC. The primer sequences used for the Δ8b mutation are as follows: forward, 5′- GACAGGACAGAAAAATTAATACCGGGAATGGAA**taa**AGCAAC**tag**CTCCCAGGTGGTACTTCTACTACACTGGAA**AGGATGACGACGATAAGTAG**-3′; reverse, 5′- TGCCATCCTTAACAGCCCGGAATGGGAGTGCTGCTTCGGGTCCAGTTCCAGTGTAGTAGAAGTACCACCTGGGAG**CAACCAATTAACCAATTCTGATTAG**-3′. The primer sequences used for the Δ9b mutation are as follows: forward, 5′- CATGGCAAGGAAGACC**taa**AATTCCCTCGAGGACAAGGCGTTCCAA**taa**ACACCAATAGCAGTCCAGATGACCAAATTGG**AGGATGACGACGATAAG****TAG**-3′; reverse, 5′- TTACCGTCACCACCACGAATTCGTCTGGTAGCTCTTCGGTAGTAGCCAATTTGGTCATCTGGACTGCTATTGGTG**CAACCAATTAACCAATTCTGATTAG**-3′. The primer sequences used for the N-D3L mutation are as follows: forward, 5′- TTTCATCTAAACGAACAAACTAAAATGTCT**cta**AATGGACCCCAAAATCAGCGAAATGCACCCCGCATTACGTTT**AGGATGACGACGATAAGTAG**-3′; reverse, 5′- ATTCTGGTTACTGCCAGTTGAATCTGAGGGTCCACCAAACGTAATGCGGGGTGCATTTCGCTGATTTTGGGGTCC**CAACCAATTAACCAATTCTGATTAG**-3′. The primer sequences used for the N-KdelA mutation are as follows: forward, 5′- TTTCATCTAAACGAACAAACTAAA**a**TGTCTGATAATGGACCCCAAAATCAGCGAAATGCACCCCGCATTACGTTT**AGGATGACGACGATAAGTAG**-3′; reverse, 5′- ATTCTGGTTACTGCCAGTTGAATCTGAGGGTCCACCAAACGTAATGCGGGGTGCATTTCGCTGATTTTGGGGTCC**CAACCAATTAACCAATTCTGATTAG**-3′. The primer sequences used for the N-double mutation are as follows: forward, 5′- TTTCATCTAAACGAACAAACTAAA**a**TGTCT**cta**AATGGACCCCAAAATCAGCGAAATGCACCCCGCATTACGTTT**AGGATGACGACGATAAGTAG**-3′; reverse, 5′- ATTCTGGTTACTGCCAGTTGAATCTGAGGGTCCACCAAACGTAATGCGGGGTGCATTTCGCTGATTTTGGGGTCC**CAACCAATTAACCAATTCTGATTAG**-3′. Mutations are highlighted in boldface lowercase letters; the A deletion is shown with a strikethrough (the primer does not contain this a residue); sequences complementary to pEP-KanS are in boldface.

### Introduction of ORF8b and ORF8b* to Δ8b MERS-CoV and MHV BACs

8b and 8b* were introduced into mouse-adapted MERS-CoV Δ8b BAC at the ORF5 region or MHV BAC at the ORF4 region by a two-step linear lambda red recombination process (36–38). The first 13 amino acids of ORF5 were retained in the BAC, immediately followed by the insert sequence. The first step removed and replaced the ORF5 sequence with GalK-Kan selection marker while the second step removed and replaced the GalK-Kan selection marker with the mutant inserts PCR amplified from the mouse-adapted MERS-CoV or mouse-adapted MERS-CoV Δ8b BAC. In brief, GalK-Kan selection marker was PCR amplified from pYD-C225 (38) and gel purified. Gel-purified GalK-Kan fragments were transformed into SW102 cells carrying the mouse-adapted MERS-CoV Δ8b BAC by electroporation, for linear lambda red recombination. Successful recombinants were selected on Kanamycin resistance culture plates. Verified recombinants carrying GalK-Kan cassette were further introduced with the corresponding inserts by electroporation for a second round of linear lambda red recombination. Successful recombinants were selected using 2-deoxy-galactose-based culture plates, and sequence identity was verified by Sanger sequencing. GalK-Kan selection markers were amplified with the following primers. For insertion to MERS-CoV ORF5, primers were as follows: forward, 5′- TTTAACGAACTATGGCTTTCTCGGCGTCTTTATTTAAACCCGTCCAGCTA**cctgttgacaattaatcatcg**-3′; reverse, 5′- GAGACTAATGGATTAGCCTCTACACGGGACCCATAGTAGCGCAGAGCTGC**ctcagcaaaagttcgattta**-3′. For insertion to MHV ORF4, primers were as follows: forward, 5′-CTCTCCTGGAAAGACAGAAAATCTAAACAATTTATAGCATTCTCATTGCTACTTTGCTCCTCTAGAGGGCAGCAAGTAGTT**cctgttgacaattaatcatcg**-3′; reverse, 5′-GTGACACCAAAGTCTCGCGTTCGGCGTAGCCAGGCGTCACTCACAAGCCAAATCTCCATGTAGCTGGTGGTGTCA**ctcagcaaaagttcgattta**-3′. Lightface capital letters represent sequence flanking the area of interest in the mouse-adapted MERS-CoV BAC or MHV BAC; sequences complementary to pYD-C225 are shown as boldface lowercase letters.

ORF8b and ORF8b* inserts were amplified with the primers below. For MERS-CoV, primers were as follows: forward, 5′- TTTAACGAACTATGGCTTTCTCGGCGTCTTTATTTAAACCCGTCCAGCTAatgccaattctacccctgcgcaaaa-3′; reverse, 5′- GAGACTAATGGATTAGCCTCTACACGGGACCCATAGTAGCGCAGAGCTGCttaagctagaggctcttgaagatgattgac-3′. For MHV, primers were as follows: forward, 5′-CTCTCCTGGAAAGACAGAAAATCTAAACAATTTATAGCATTCTCATTGCTACTTTGCTCCTCTAGAGGGCAGCAAGTAGTTatgccaattctacccctgcgcaaaa-3′; reverse, 5′-GTGACACCAAAGTCTCGCGTTCGGCGTAGCCAGGCGTCACTCACAAGCCAAATCTCCATGTAGCTGGTGGTGTCAttaagctagaggctcttgaagatgattgac-3′. The capital letters represent sequence of the area where recombination takes place (flanking ORF5 or ORF4 for MHV), while the lowercase letters represent sequence complementary to ORF8b for PCR amplification with either mouse-adapted MERS-CoV (for Δ8b-8b) or mouse-adapted MERS-CoV Δ8b (for Δ8b-8b*) BAC as template.

### Rescue of BAC-derived virus and virus propagation

Two micrograms of the indicated MERS-CoV or SARS-CoV-2 BACs was transfected into Huh-7 cells (ATCC) or Vero E6 cells, respectively, with Lipofectamine 3000 (Invitrogen) in a six-well plate according to the manufacturer’s protocol. Cells were monitored daily for cytopathic effects (CPE). Cultures were harvested when CPE was >50% by freezing at −80°C. MERS-CoV were further passaged in Huh-7 cells in DMEM supplemented with 10% FBS. SARS-CoV-2 were propagated in Calu-3 cells. Virus titer was determined by plaque assay as described in the previous section. Huh-7 cells were infected with MERS-CoV, and RNA was isolated for RT-PCR. PCR products were sent for Sanger sequencing to verify that mutations were correctly introduced. SARS-CoV-2 sequences were verified as previously described (21).

### Proteomics sample preparation

Each sample was digested in an S-Trap micro spin column (ProtiFi, USA) according to the manufacturer’s instructions. Calu-3 cell pellets were lysed with 25 mL 5% SDS in 50 mM TEAB buffer containing protease inhibitors (Pierce). The protein concentration of each sample was measured using a BCA assay (Pierce); 50 µg total protein was used for further analysis. Proteins in each sample were reduced by adding 4.5 mM (final concentration) dithiothreitol and incubated for 15 min at 55°C. Proteins were then alkylated by the addition of 10 mM (final concentration) iodoacetamide for 10 min at room temperature in the dark. Each sample was then acidified by adding phosphoric acid to a 2.5% final concentration. The samples were mixed with 6× volumes of binding buffer (90% methanol; 100 mM TEAB) and loaded onto the S-trap filter and centrifuged at 4,000 × *g* for 30 s. The samples were washed three times with wash solution (90% methanol; 100 mM TEAB) before digestion with 5 µg trypsin (Promega) (1/10, wt/wt) overnight at 37°C.

The S-trap filters containing digested peptides were then rehydrated for 30 min at room temperature with 40 µL of elution buffer 1 (50 mM TEAB in water). Subsequent elution steps of 40 µL of elution buffer 2 (0.2% trifluoroacetic acid [TFA] in water) followed by 40 µL elution buffer 2 (50% acetonitril [ACN] in water) completed the peptide elution. The peptide eluates were pooled and de-salted with reverse-phase C18 OMIX tips (Pierce), all according to the manufacturer’s specifications before proceeding to LC-MS/MS analysis.

### LC-MS/MS and data analysis

Peptides were analyzed by LC-MS/MS using a nanoElute coupled to a timsTOF Pro2 Mass Spectrometer (Bruker Daltonics). Samples were loaded on a capillary C18 column (25 cm length, 75 µm inner diameter, 1.6 µm particle size, and 120 Å pore size; IonOpticks). The flow rate was kept at 300 nL/min. Solvent A was 0.1% FA in water, and solvent B was 0.1% FA in ACN. The peptides were separated on a 54.5-min analytical gradient from 2% to 35% of Solvent B for a total of a 60-min run time. The timsTOF Pro2 was operated in the PASEF mode. MS and MS/MS spectra were acquired from 100 to 1,700 m/z. The inverse reduced ion mobility 1/K_0_ was set to 0.60–1.60 V·s/cm^2^ over a ramp time of 100 ms. Data-dependent acquisition was performed using 10 PASEF MS/MS scans per cycle with a near 100% duty cycle.

Data analysis was performed with Fragpipe (version 18.0) using the MSFragger (version 3.6) search engine configured with Philosopher (version 4.6.0) and IonQuant (version 1.8.9) with default search settings for LFQ-MBR (match-between-runs) workflow including a false discovery rate set at 1% on both the peptide and protein level. Spectra were compared against *Homo sapiens* (UniProt ID UP000005640) protein database containing 81,837 human sequences as well as proteins from the SARS-CoV-2 protein database (UniProt ID UP000464024) containing 17 viral protein sequences. For both searches, a mass tolerance for precursor ions was set at a range of −20 ppm to 20 ppm with a mass tolerance for fragment ions at 20 ppm. A maximum of two missed cleavages was set for the shotgun searches. Carbamidomethylation of cysteine residues was set as a fixed modification while variable modifications were set to oxidation of methionine and acetylation of protein N-termini. Only proteins with at least one unique or razor peptide were retained in the shotgun search. One replicate from rSARS2_MA30_-infected samples was excluded as being an outlier in intensity.

### RNA sequencing and analysis

Stranded RNAseq libraries were constructed using the Kapa mRNA Hyper Prep Kit (Roche, Indianapolis, IN). Briefly, the total RNA was quantitated by Qubit (Life Technologies, Grand Island, NY) and assessed for quality with a Fragment Analyzer (Agilent Technologies, Santa Clara, CA). Next, polyA+ RNA was selected from 200 ng of total RNA per sample. PolyA+ RNA was fragmented for 4 min at 94°C, and then, first-strand cDNA was synthesized with a random hexamer and SuperScript II (Life Technologies). Double-stranded DNA was blunt-ended, 3′-end A-tailed, and ligated to a universal adaptor. The adaptor-ligated double-stranded cDNA was amplified by PCR for 12 cycles with Twist Unique Dual-Index Primers (Twist Bioscience, San Francisco, CA). The final libraries were quantitated on Qubit, and the average size was determined on the Fragment Analyzer and diluted to 5 nM final concentration. The 5 nM dilution was further quantitated by qPCR on a BioRad CFX Connect Real-Time System (Bio-Rad Laboratories Inc., CA). The final stranded RNAseq library pool was sequenced on one lane of an Illumina NovaSeq 6000 S4 flowcell as paired reads with a 150-nt length. The run generated .bcl files which were converted into adaptor-trimmed demultiplexed fastq files using bcl2fastq v2.20 Conversion Software (Illumina, CA).

The adapter sequences and poly-A tails sequencing reads were removed by cutadapt (39). The adapter-removed sequencing reads were then aligned to NC_019843 sequence to remove reads coming from the MERS-CoV genome. Only aligned reads with less than 10% of their sequences aligned were removed. The same read removal process was subsequently done with mouse ribosomal DNA complete repeat unit (BK000964.3) to remove reads coming from rRNA. Transcript abundances were then calculated by Kallisto (40) with the sequencing reads that were not filtered away from the above filtering steps. Differential expression analysis was done with DESeq2 (41). All analysis codes used for data processing are deposited on GitHub at https://github.com/wckdouglas/MERS-CoV-mouse.

### Sequence alignment and MTS prediction

The internal protein sequences of MERS-CoV (NC_019843), SARS-CoV (NC_004718), and SARS-CoV-2 (NC_045512) were imported from NCBI. Sequences were aligned with Clustal Omega (https://www.ebi.ac.uk/Tools/msa/clustalo/). Results were analyzed and images were exported with Jalview.

Sequence of MERS-CoV ORF8b serially truncated from the N-terminus was submitted to the TargetP-2.0 server (https://services.healthtech.dtu.dk/service.php?TargetP-2.0) for prediction of encoding an internal MTS (26, 27).

### Structure prediction

AlphaFold Colab (24, 42), a simplified version of AlphaFold v2.3.1 204, was used to generate models of the MERS ORF8b protein. The amino acid sequence of MERS ORF8b, “MPILPLRKMLGIGGDRTEKLIPGMELSNWLPGGTSTTLELDPKQHSHSGLLRMASFGSMKMAPLMLLQLLGRGTLTMIQLLLHNSRPVLSFLKTSTLRGLEAIVNHLQEPLA,” was used as input. Default parameters were used.

### Statistical analysis

A Student’s *t*-test was used to analyze differences in mean values between groups. All results are expressed as mean ± SEM except for virus titers and viral RNA levels where data are represented as geometric mean ± geometric SD. *P*-values of <0.05 were considered statistically significant. *P < 0.05, **P < 0.01, and ***P < 0.001. Differences in mortality were analyzed using Kaplan-Meier log-rank survival tests.

## Data Availability

Raw sequencing data generated in this study have been deposited in the NIH Short Read Archive under accession number BioProject PRJNA946177. Mass spectrometry proteomics data have been deposited to the ProteomeXchange Consortium via the PRIDE repository with the data set identifier PXD042023.

## SUPPLEMENTAL MATERIAL

Supplemental figures mbio.02476-23-s0001.pdf Fig. S1 to 7.

Legends mbio.02476-23-s0002.docx Legends for Fig. S1 to S7.

Table S1 mbio.02476-23-s0003.xlsx Peptides.

ASM does not own the copyrights to Supplemental Material that may be linked to, or accessed through, an article. The authors have granted ASM a non-exclusive, world-wide license to publish the Supplemental Material files. Please contact the corresponding author directly for reuse.
