# Supplementary material for: Contrasting roles of MERS-CoV and SARS-CoV-2 internal proteins in pathogenesis in mice
Source: mBio. 2023 Oct 26;14(6):e02476-23. doi: 10.1128/mbio.02476-23 (PMC10746224; doi:10.1128/mbio.02476-23)
Supplement: Supplemental figures — Fig. S1 to 7. [file mbio.02476-23-s0001.pdf]

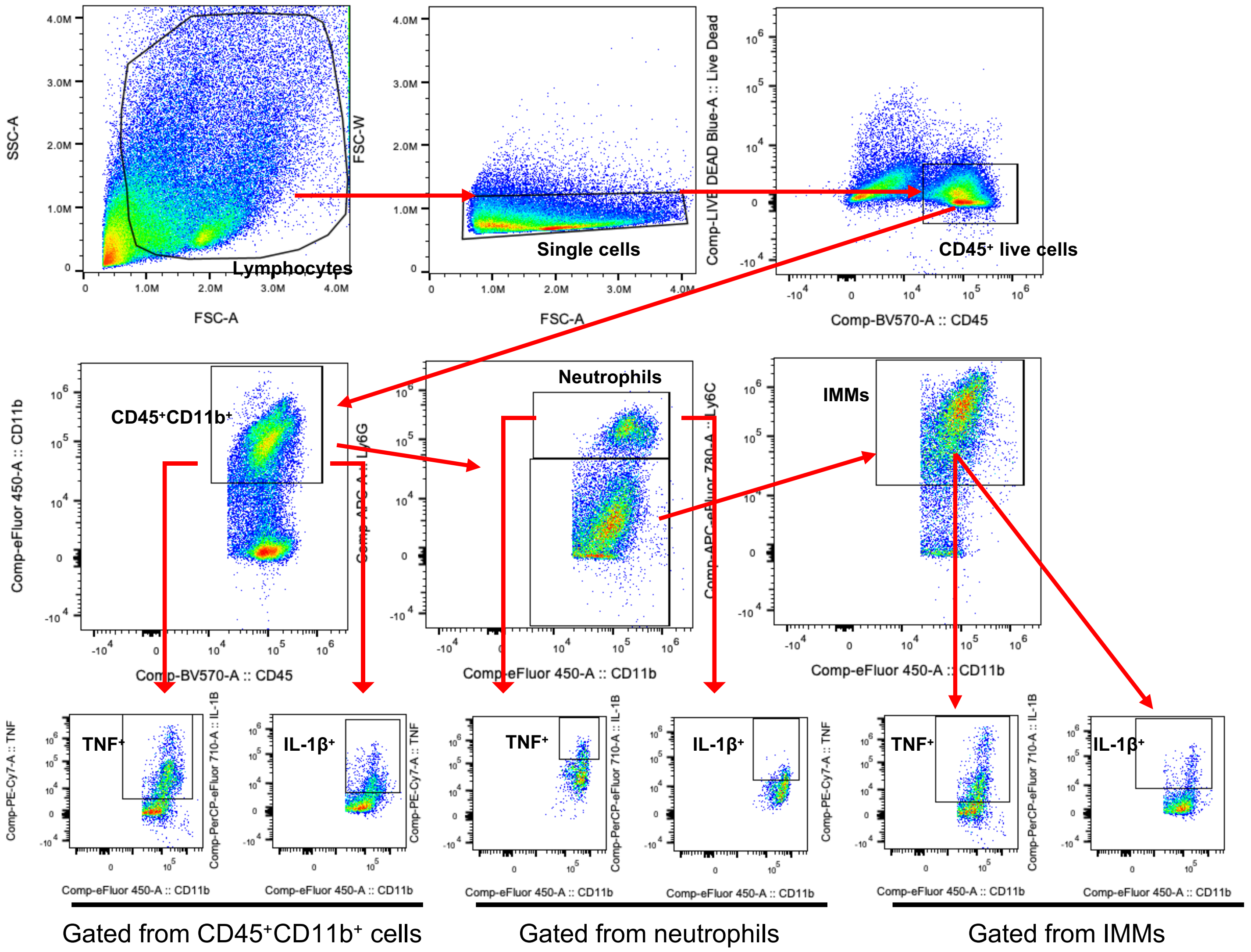

Lymphocytes
Single cells
CD45⁺ live cells
CD45⁺CD11b⁺
Neutrophils
IMMs
TNF⁺
IL-1β⁺
SSC-A
FSC-A
FSC-W
Comp-LIVE DEAD Blue-A :: Live Dead
Comp-BV570-A :: CD45
Comp-eFluor 450-A :: CD11b
Comp-APC-A :: Ly6G
Comp-APC-eFluor 780-A :: Ly6C
Comp-PE-Cy7-A :: TNF
Comp-PerCP-eFluor 710-A :: IL-1B
Gated from CD45⁺CD11b⁺ cells
Gated from neutrophils
Gated from IMMs

Figure S1

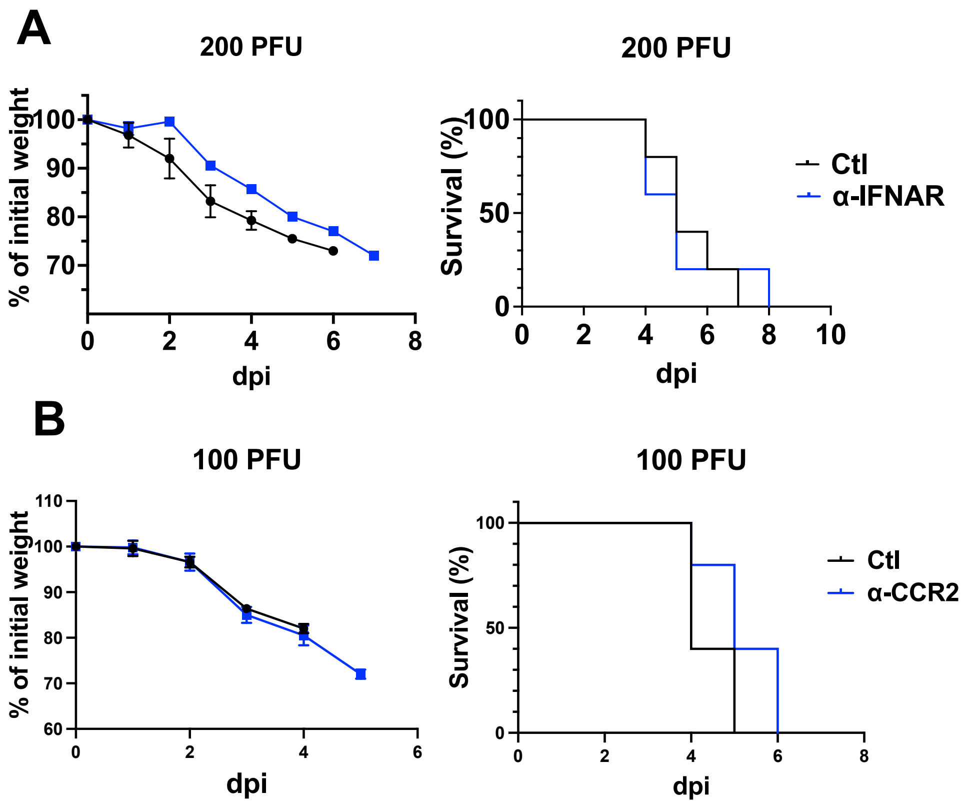

A
200 PFU
% of initial weight
100
90
80
70
0
2
4
6
8
dpi
200 PFU
Survival (%)
100
50
0
0
2
4
6
8
10
dpi
Ctl
α-IFNAR
B
100 PFU
% of initial weight
110
100
90
80
70
60
0
2
4
6
dpi
100 PFU
Survival (%)
100
50
0
0
2
4
6
8
dpi
Ctl
α-CCR2

Figure S2

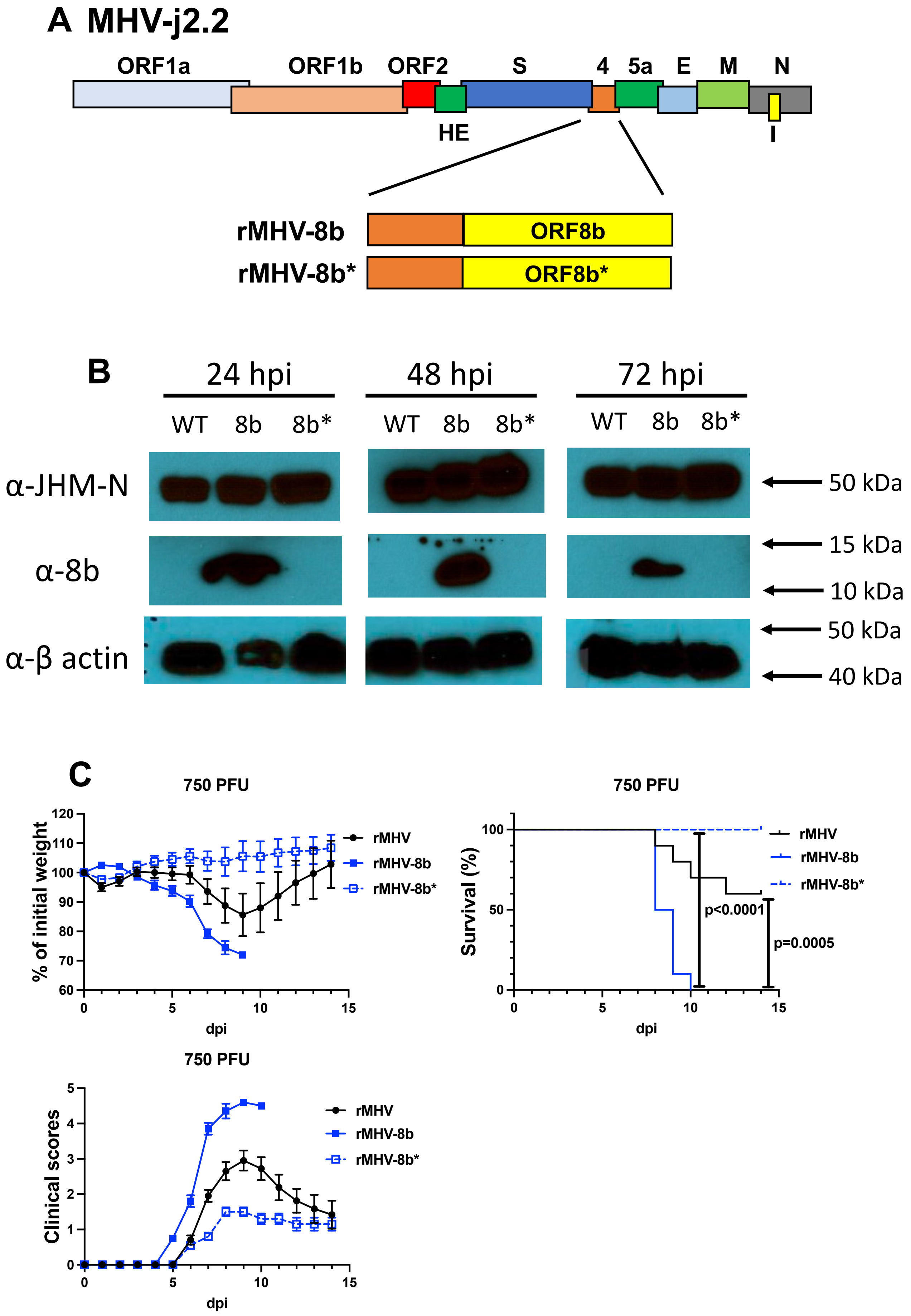
A MHV-j2.2
ORF1a
ORF1b
ORF2
S
4
5a
E
M
N
HE
I
rMHV-8b
ORF8b
rMHV-8b*
ORF8b*
B
24 hpi
48 hpi
72 hpi
WT 8b 8b*
α-JHM-N
α-8b
α-β actin
50 kDa
15 kDa
10 kDa
50 kDa
40 kDa
C
750 PFU
% of initial weight
dpi
rMHV
rMHV-8b
rMHV-8b*
Survival (%)
p<0.0001
p=0.0005
Clinical scores

Figure S3

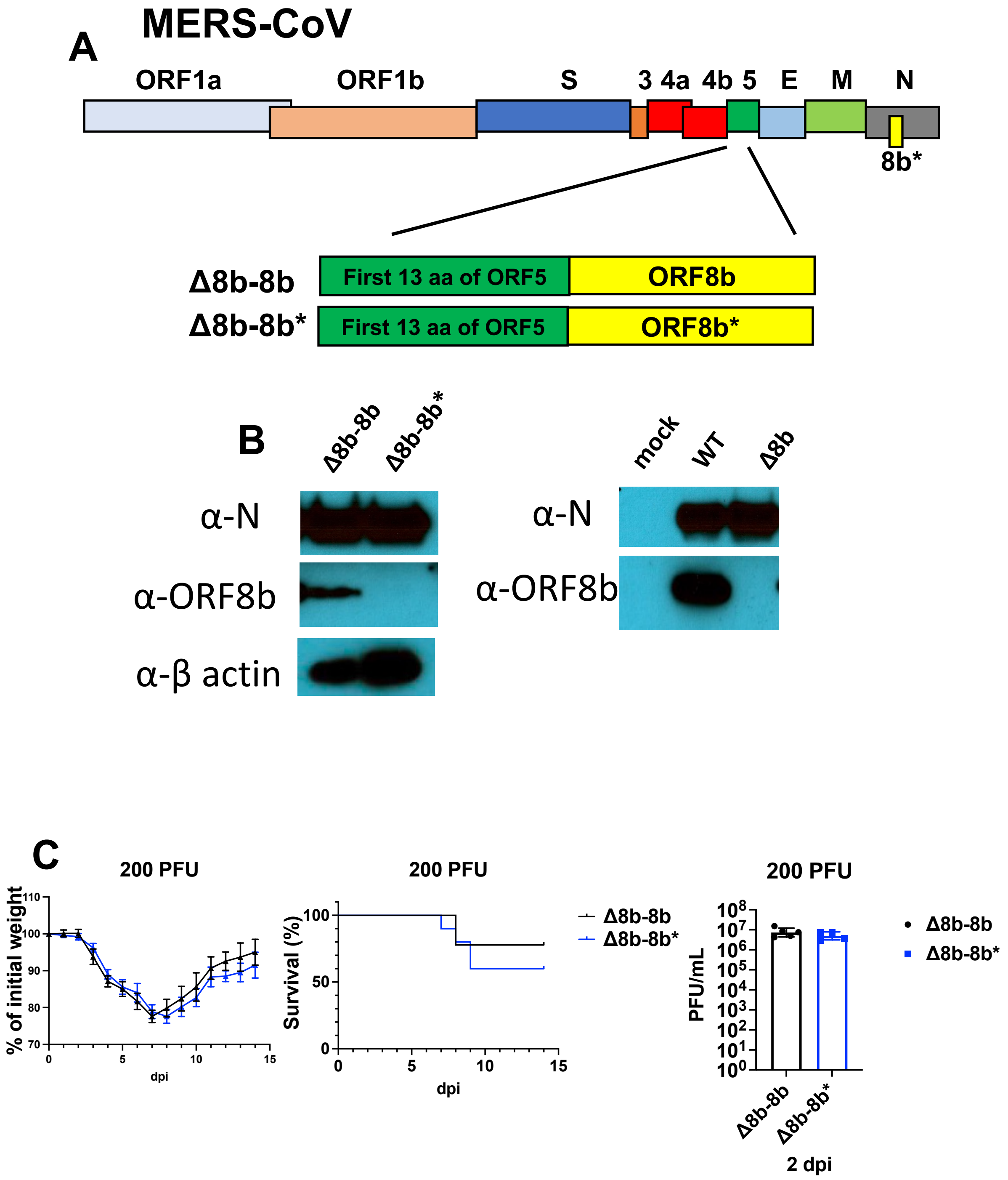
A
MERS-CoV
ORF1a
ORF1b
S
3
4a
4b
5
E
M
N
8b*
Δ8b-8b
First 13 aa of ORF5
ORF8b
Δ8b-8b*
First 13 aa of ORF5
ORF8b*
B
Δ8b-8b
Δ8b-8b*
α-N
α-ORF8b
α-β actin
mock
WT
Δ8b
α-N
α-ORF8b
C
200 PFU
% of initial weight
dpi
200 PFU
Survival (%)
dpi
Δ8b-8b
Δ8b-8b*
200 PFU
PFU/mL
Δ8b-8b
Δ8b-8b*
2 dpi
Δ8b-8b
Δ8b-8b*

Figure S4

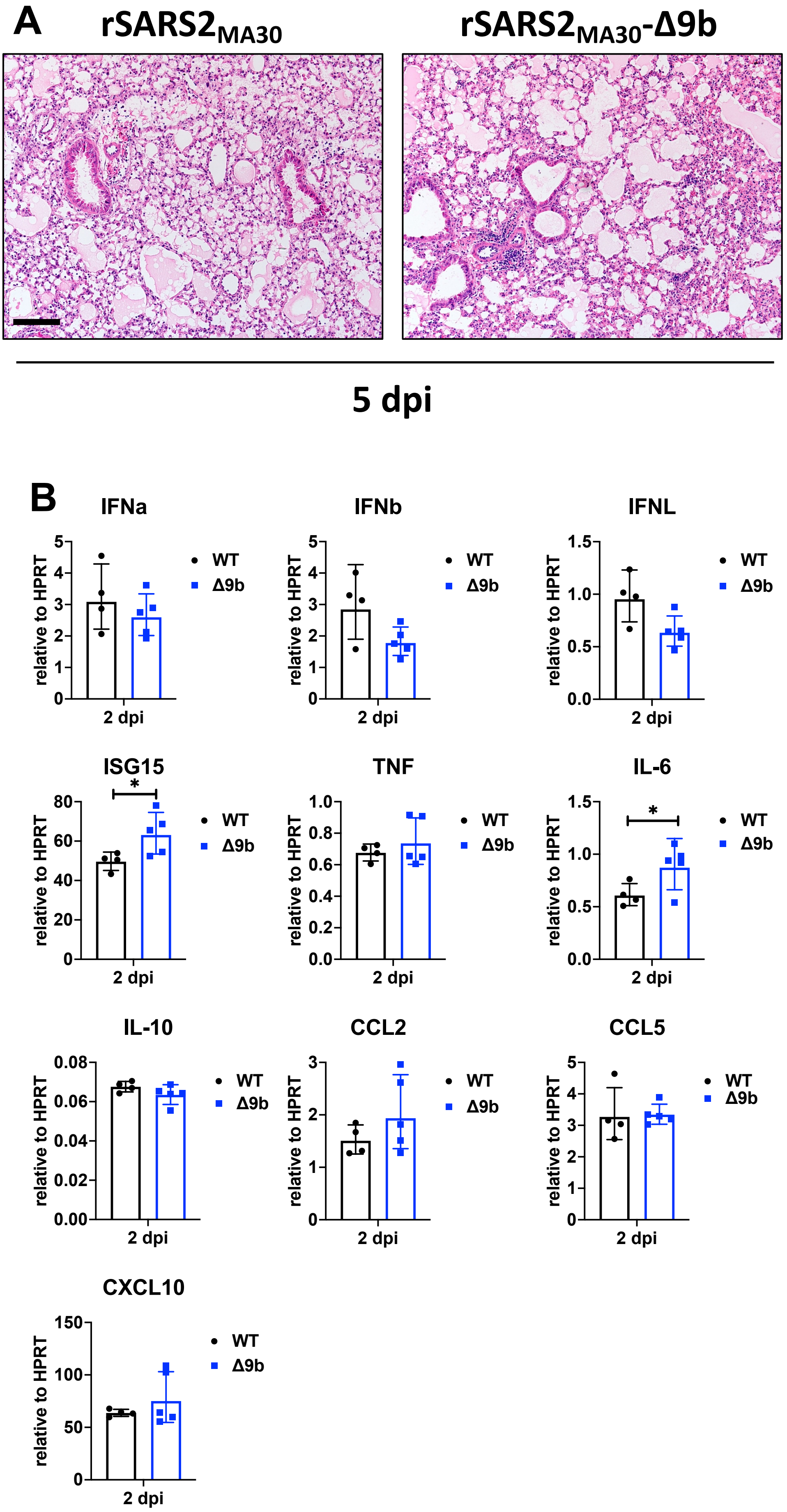
A
rSARS2MA30
rSARS2MA30-Δ9b
5 dpi
B
IFNa
IFNb
IFNL
ISG15
TNF
IL-6
IL-10
CCL2
CCL5
CXCL10
relative to HPRT
2 dpi
WT
Δ9b
*

Figure S5

A

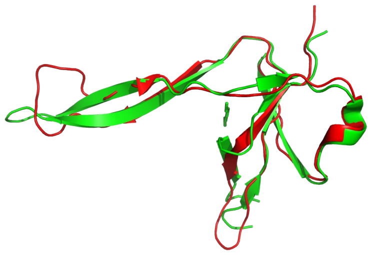

2CME (red) vs 7YE8 (green)

B

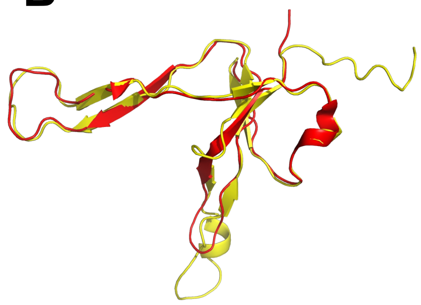

2CME (red) vs SARS-CoV 9b prediction (yellow)

C

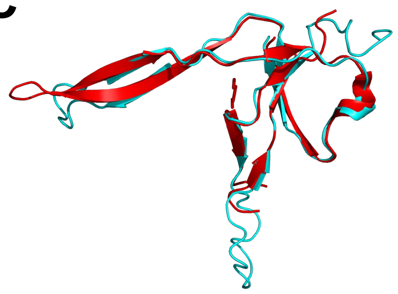

7YE8 (red) vs SARS-CoV-2 9b prediction (teal)

Figure S6

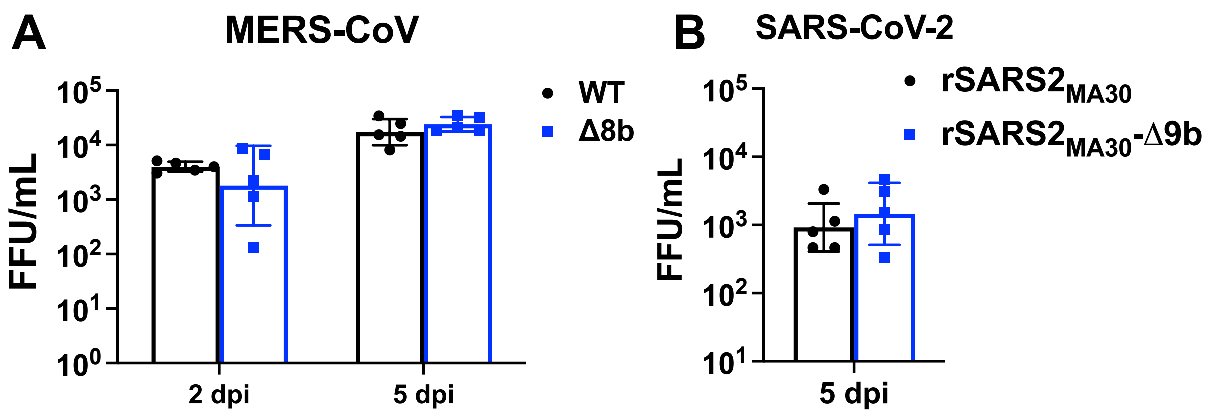
A
MERS-CoV
FFU/mL
10^5
10^4
10^3
10^2
10^1
10^0
WT
Δ8b
2 dpi
5 dpi
B
SARS-CoV-2
FFU/mL
10^5
10^4
10^3
10^2
10^1
rSARS2MA30
rSARS2MA30-Δ9b
5 dpi

Figure S7
