# Supplementary material for: Contrasting roles of MERS-CoV and SARS-CoV-2 internal proteins in pathogenesis in mice
Source: mBio. 2023 Oct 26;14(6):e02476-23. doi: 10.1128/mbio.02476-23 (PMC10746224; doi:10.1128/mbio.02476-23)
Supplement: Legends — for Fig. S1 to S7. [file mbio.02476-23-s0002.docx]

**Fig. S1 Gating strategy for CD11b^+^ cells, neutrophils and IMMs**.

**Fig. S2 Blockade of IFN signaling or depletion of IMMs did not improve clinical disease after rMERS_MA_-Δ8b infection.**

(A) Percent of initial weight (left panel) and survival (right panel) of hDPP4-KI mice infected with sublethal dose of rMERS_MA_-Δ8b. Infected mice were treated with 750 μg of control antibody (Ctl, black line) or anti-IFNAR antibody (α-IFNAR, blue line) by intraperitoneal injection at 1 dpi. (B) Percent of initial weight (left panel) and survival (right panel) of hDPP4-KI mice infected with sublethal dose of rMERS_MA_-Δ8b. Infected mice were treated with 25 μg of control antibody (Ctl, black line) or anti-CCR2 antibody (α-CCR2R, blue line) by intraperitoneal injection at 2 and 4 dpi.

**Fig. S3 Introduction of MERS-CoV protein 8b into MHV enhanced pathogenicity**.

(A) Schematic diagram showing the introduction of MERS-CoV ORF8b (rMHV-8b) and the version of MERS-CoV ORF8b with two stop codons (rMHV-8b*) into the ORF4 region of MHV (B) 17Cl-1 cells were infected with the indicated viruses at an MOI of 0.01 and total protein were harvested at the specified time point post-infection. N protein and ORF8b expression were detected by using antibodies targeting endogenous expression. (C) Percent of initial weight (top left), survival (top right) and clinical score (bottom) of C57BL/6 mice infected with 750 PFU of recombinant rMHV (black solid line), rMHV-8b (blue solid line) or rMHV-8b* (blue dashed line).

**Fig. S4 Introduction of MERS-CoV 8b to ORF5 did not result in attenuation.**

(A). Schematic diagram showing the introduction of MERS-CoV ORF8b (Δ8b-8b) and the defective version of MERS-CoV ORF8b with two stop codons (Δ8b-8b*) into the ORF5 region in the background of the rMERS_MA_-Δ8b virus. (B). Huh-7 cells were infected with the indicated viruses at an MOI of 0.01. Cells were harvested at 48 hpi for detecting N and MERS-CoV ORF8b expression using indicated antibodies. (C). Percent of initial weight (left), survival (middle) and infectious virus titer (right) of hDPP4-KI mice infected with 100-200 PFU Δ8b-8b (black) or Δ8b-8b* (blue).

**Fig. S5 Histopathology and inflammatory responses after rSARS2_MA30_ or rSARS2_MA30_-Δ9b infection**.

(A) Representative H&E stain of lungs of C57BL/6 mice infected with 1000 PFU of rSARS2_MA30_ or rSARS2_MA30_-Δ9b at 5 dpi. Scale bar = 142 μm. (B) C57BL/6 mice infected with 1000 PFU of rSARS2_MA30_ or rSARS2_MA30_-Δ9b were harvested at the 2 dpi. Lungs were homogenized and RNA was isolated for measurement of cytokine and chemokine levels by RT-qPCR

**Fig. S6 Structural comparison of SARS-CoV and SARS-CoV-2 protein 9b.**

Superimposed images of (A) SARS-CoV and SARS-CoV-2 ORF9b, (B) published SARS-CoV ORF9b and AlphaFold-predicted SARS-CoV ORF9b and (C) published SARS-CoV-2 ORF9b and AlphaFold-predicted SARS-CoV-2 ORF9b.

**Fig. S7 The Internal proteins of Betacoronaviruses have minimal effects in virus replication in the upper respiratory tract of infected animals.**

(A) hDPP4-KI mice were intranasally infected with 100-200 PFU of rMERS_MA_ or rMERS_MA_-Δ8b. Infectious virus titers in the nasal turbinates were determined at 2 and 5 dpi by plaque assay. (B) C57BL/6 mice were intranasally infected with 1000 PFU of rSARS2_MA_ or rSARS2_MA_-Δ9b. Infectious virus titers in the nasal turbinates at 2 dpi are shown. Each point represents data obtained from an individual mouse.
